# Effects of Hemp-Derived Cannabidiol Supplementation on Blood Variables, Carcass Characteristics, and Meat Quality of Goats

**DOI:** 10.3390/ani14121718

**Published:** 2024-06-07

**Authors:** Tanom Tathong, Supawut Khamhan, Salinee Soisungwan, Chirasak Phoemchalard

**Affiliations:** 1 Department of Food Technology, Faculty of Agriculture and Technology, Nakhon Phanom University, Nakhon Phanom 48000, Thailand; salinee.soi@npu.ac.th; 2 That Phanom College, Nakhon Phanom University, Nakhon Phanom 48110, Thailand; es1414@npu.ac.th; 3 Department of Agriculture, Mahidol University, Amnatcharoen Campus, Amnatcharoen 37000, Thailand; chirasak.pho@mahidol.edu; 4 Excellence Center on Agriculture and Food for Health, Mahidol University, Amnatcharoen Campus, Amnatcharoen 37000, Thailand

**Keywords:** cannabidiol, hemp, nutrition, animals, meat quality

## Abstract

**Simple Summary:**

Dietary supplements can affect animal growth, product quality, and various physiological factors. This study investigated the effects of supplementing hemp-derived cannabidiol (CBD) oil in the diet of goats. CBD, when included at higher levels (0.2–0.3 mL), led to improved meat quality but also altered volatile compound profiles. Additionally, CBD supplementation affected some blood variables related to immunity, metabolism, and homeostasis.

**Abstract:**

Stress experienced by animals during pre-mortem management handling significantly affects both their welfare and the quality of the meat produced. Using hemp-derived CBD may offer several benefits in alleviating this issue. In this study, we investigated the effects of hemp-derived CBD supplementation on blood variables, growth performance, carcass characteristics, and meat quality in goats. Sixteen crossbred Boer goats were divided into four groups receiving a basal diet supplemented with 0 (control), 0.1, 0.2, or 0.3 mL CBD/30 kg body weight over 90 days. Although growth, carcass characteristics, and pH remained unaffected, CBD supplementation influenced several blood variables. Specifically, dietary CBD at 0.1–0.3 mL increased white blood cell (WBC) counts, while 0.3 mL CBD increased serum total protein, globulin, sodium, and carbon dioxide levels, potentially affecting protein metabolism and electrolyte balance. Over time, significant changes were noted in hematological profiles, kidney markers, protein profiles, and some electrolytes, indicating physiological adaptations. Regarding meat quality, supplementation with 0.2–0.3 mL of CBD linearly improved color redness and stability; moreover, CBD supplementation improved tenderness and textural properties, resulting in a softer meat texture. However, analysis using an E-nose indicated increased ammonia and organic solvent vapors in meat from the higher CBD groups. This study concluded that CBD supplementation up to 0.3 mL of CBD/30 kg body weight beneficially modulated blood biomarkers, meat color, and tenderness without adverse impacts on growth or carcass characteristics in goats.

## 1. Introduction

Goats are a vital livestock species worldwide, valued for their meat, milk, and fiber [1]. The demand for goat meat has increased significantly, driven by its nutritional benefits, such as high levels of protein, vitamins, and minerals, and its growing popularity among diverse consumer groups, particularly in Muslim nations [2,3,4]. However, stress in farm animals is a significant concern due to its adverse effects on both animal welfare and meat safety [5]. The stress that animals experience during pre-mortem management can have a significant impact on their welfare and the quality of meat produced [1]. Among the various strategies for mitigating stress in farm animals, the use of hemp and its by-products has emerged as the most promising intervention for promoting stress reduction and improving animal welfare [6,7].

Despite hemp’s potential benefits, there is limited information on the effects of hemp-derived cannabidiol (CBD) on animal growth, carcass characteristics, and meat quality. The incorporation of hemp by-products into livestock feed has not significantly affected growth performance, carcass characteristics, or most meat quality [6,8,9]; however, certain hematological indices associated with liver function, renal physiology, and inflammatory status have exhibited alterations in response to CBD derived from hemp in a dietary regimen [7,8]. In livestock, hemp and its derivatives offer a range of potential nutritional (essential amino acids, fatty acids, minerals, vitamins, and fiber), health (reducing stress), and therapeutic (reducing pain and anti-inflammatory) benefits [10,11,12]. Similarly, cannabidiolic acid (CBDA)-rich hemp could be a viable feed component for promoting welfare and reducing stress-related issues in livestock [7]. Additionally, preclinical studies indicate that CBD has anti-inflammatory, antioxidant, analgesic, and antidepressant properties in various animal models, indicating its potential therapeutic value in maintaining homeostasis and promoting animal health and welfare [11,12].

Although hemp and its constituent compounds may provide potential nutritional, therapeutic, and health-promoting advantages for livestock species, the specific effects of supplementing pure CBD oil on production traits and meat quality in goats remain largely unknown. Therefore, this study aimed to evaluate the potential effects of graded levels of hemp-derived CBD supplementation on the blood variables, growth performance, carcass characteristics, and meat quality of goats. The results of this study could contribute to the development of innovative and sustainable feeding strategies to improve goat welfare and meat quality while meeting consumer demands for safe and nutritious animal-sourced foods.

We hypothesized that CBD administration would positively influence blood biochemistry, improve carcass characteristics, and enhance meat quality while maintaining consistent growth rates.

## 2. Materials and Methods

This research was conducted on the project “Development of Hemp Production and Product for Growth and Economic Animal Health in Greater Mekong Subregion”. All animal-related experimental procedures were approved by the Animal Ethics Committee of Nakhon Phanom University (Approval No: NPU011/2566), which adheres to strict standards for animal welfare and ethical research practices. 

### 2.1. Animal and Experimental Design

The goats used in the experiment were sourced from local farms in the Mukdahan and Nakhon Phanom provinces in Thailand. Before the trial, the goats underwent a one-month adaptation period at the experimental facility, during which they were provided with appropriate housing and husbandry. During this adaptation phase, the goats were fed a balanced commercial diet at 0.5% of their body weight, formulated with 12% crude protein, 2% crude fat, 13% crude fiber, and 13% moisture content. They were also provided ad libitum access to water and rice straw for roughage sources. The nutrient composition of rice straw in this study included 11% moisture, 3% crude protein, 2% crude fat, 36% crude fiber, and 6% ash. After a 30-day acclimatization period, 16 crossbred male Boer goats (average body weight: 14.10 ± 2.81 kg; age: six months old) were randomly assigned to four groups of four animals, each using a completely randomized design (CRD). The control group (T1) received only the basal diet with roughage, while the treatment groups received the basal diet supplemented with cannabidiol (CBD, 96% concentration) at levels of 0.1 (T2), 0.2 (T3), and 0.3 (T4) mL/30 kg body weight. Animals in the treatment groups received a daily oral administration of CBD oil softgel capsules. This experiment was conducted for 90 days.

### 2.2. Blood Variables

To evaluate the potential effect of CBD on blood variables, jugular venous blood samples were collected from each experimental group of goats at three different time points: on the first day before CBD supplementation (Day 0; D0), mid-trial (Day 45; D45), and the final day (Day 90; D90) of the experiments. Blood collection was carried out before the morning feeding. These collected blood samples of approximately 5 mL were transferred to two separate tubes: a lithium heparin tube (2.5 mL) and an ethylenediaminetetraacetic acid (EDTA) tube (2.5 mL). They were immediately mixed, stored in an ice box, transferred to a commercial laboratory, and then analyzed for a comprehensive panel of hematological and biochemical variables. Hematological assessments included total white blood cells (WBCs), hemoglobin (Hb) concentration, hematocrit (Hct) value, and differential leukocyte counts for neutrophils, lymphocytes, eosinophils, and monocytes. Biochemical analyses targeted markers of renal function (blood urea nitrogen, BUN; creatinine), protein metabolism (total protein, albumin, and globulin), hepatobiliary function (alkaline phosphatase), electrolyte balance (sodium, potassium, chloride, and carbon dioxide), and mineral homeostasis (calcium, magnesium, and phosphorus). All hematological and biochemical variables were quantified using an automated hematology analyzer (Sysmex XT-2000iV™, Goerlitz, Germany) following strict quality control measures and adherence to the manufacturer’s instructions.

### 2.3. Measurements of Growth Performance and Carcass and Non-Carcass Characteristics

Data collection included initial body weight (kg), final body weight (kg), weight gain (kg), and average daily gain (g). At the end of the trial period, the goats fasted for 12 h before being slaughtered, after which the carcasses were conventionally dressed. The hot carcass weight (kg), dressing percentage, and rib-eye area (cm^2^) were recorded. Carcass composition was evaluated by separating and weighing individual primal cuts as percentages of hot carcass weight (%CW), including neck, shoulder, and forelegs (combined); rack, loin, tenderloin, leg, and hind legs (combined); and the breast, flank, sternum, and trimmings. Non-carcass characteristics were quantified as percentages of final body weight (%FBW), including the head, tail, foreshanks, hind shanks, liver, heart, lungs, and trachea (combined); kidneys, spleen, penis, and testes (combined); stomach, intestines, internal fat depots, and the collective weight of blood and gastrointestinal contents.

### 2.4. Measurements of Meat Quality

Approximately 1 kg of meat samples (*longissimus thoracis et lumborum*) were immediately collected following slaughter, sealed in a plastic bag, stored on ice at 2–3 °C, and then transported to the laboratory for further quality analysis. Meat samples were chilled at 4 °C in a refrigerator until 24 h post-mortem. Meat quality variables—including pH, color, water-holding capacity, and texture properties—were measured. The ultimate pH (pH_u_) was determined 24 h post-mortem by inserting a Hanna HI99163 pH meter equipped with an FC232D probe (Hanna Instruments, Inc., Laval, QC, Canada) into the muscle at three sampling points and then calculating the mean value. Surface color was objectively quantified in triplicate using a Minolta CR-400 Chroma Meter (Konica Minolta Business Solutions Company Limited, Bangkok, Thailand) after a 15-min blooming period at room temperature. Color measurements were taken in triplicate, capturing values for lightness (L*), redness (a*), yellowness (b*), C* (chroma), and h* (hue angle), and averaged from three distinct locations on the muscle surface, following the American Meat Science Association (AMSA) methodology [13]. Chroma represents color intensity and takes on values from zero to one; higher values represent vivid colors, whereas hue angle shows the color tone and ranges from purplish to yellowish red in meat to distinguish shades [13]. Drip loss was assessed by suspending a 35 g loin sample in a bag at 4 °C for 48 h, then calculating the weight loss percentage. Cooking loss was evaluated using the following protocol [14]. Forty-five-gram loin slices were weighed before and after vacuum-bag cooking at an internal temperature of 72 °C in an 80 °C water bath for 15 min, followed by chilling overnight at 4 °C. Shear force measurements were performed on cooked cores excised parallel to the muscle fiber orientation, using a Brookfield CT3 Texture Analyzer fitted with a Warner-Bratzler shear blade. The instrumental settings were as follows: a trigger force of 20 g, deformation distance of 35 mm, crosshead speed of 5 mm/s, and a sample size of 0.5 cm^3^. Texture profile analysis (TPA) was conducted on cooked loin meat samples using a Brookfield CT3 Texture Analyzer, following the methodology described by Bourne [15]. The TPA specifications included a trigger force of 5 g, deformation distance of 5 mm, crosshead speed of 4 mm/s, travel distance of 20 mm, and a sample size of 1 × 1 × 1 cm.

### 2.5. Proximate Composition of the Meat

The chemical composition of meat from the *longissimus thoracis et lumborum* muscles, comprising moisture, protein, fat, and ash content, was analyzed in triplicate, adhering to the AOAC methodology [16]. 

### 2.6. Electronic Nose Analysis

The analysis of volatile compounds from goat meat samples was facilitated by an electronic nose system (Electronic Nose Company Limited, Bangkok, Thailand) incorporating an array of eight gas sensors: TGS 816, TGS 2600, TGS 823, TGS 2603, TGS 826, TGS 2610, TGS 2620, and TGS 2444. For each analysis, approximately 10 g of meat from the *longissimus thoracis et lumborum* muscle was placed in a 100 mL Duran bottle, and the headspace volatiles were sampled with the electronic nose. The measurement protocol consisted of five consecutive cycles, each with a 120-s reference phase to establish a baseline, followed by a 30-s acquisition phase to analyze the sample headspace, and a 60-s purge phase to eliminate residual volatiles between samples. The responses of the individual gas sensors were recorded using CIMS NOSE 2.0 software and quantified as relative percentage changes compared to the reference air, following the methodologies described in references [17,18].

### 2.7. Statistical Analysis

Data on hematological variables, growth performance, carcass characteristics, and meat quality of goats supplemented with CBD were subjected to statistical analysis following a completely random design (CRD) model. Differences among treatment group means were evaluated for significance (*p* < 0.05) using Duncan’s New Multiple Range Test, which was facilitated by the open-source software package Rstudio 2023.06.1 Build 524 version [19,20]. An orthogonal polynomial analysis was also used to evaluate the trend between varying levels of CBD supplementation. All analyses are presented as least squares mean values. 

## 3. Results

Table 1 presents the effects of CBD supplementation on serum hematological and biochemical variables in goats. According to Table 1, the treatment × time interaction was not significant for any of the variables; however, there was a significant effect of treatment on WBC counts (*p* < 0.05), with the T2 (0.1 mL) and T4 (0.3 mL) groups exhibiting higher levels than the T1 (control) and T3 (0.2 mL) groups. The total protein and globulin concentrations were significantly higher (*p* < 0.05) in the T4 group compared to other treatments, while albumin levels remained unaffected (*p* > 0.05). Furthermore, treatment had a significant influence (*p* < 0.05) on serum sodium and carbon dioxide levels but not on other variables (*p* > 0.05), such as alkaline phosphatase, potassium, chloride, calcium, magnesium, or phosphorus concentrations.

The timing of blood sampling (D0, D45, and D90) had significant effects on several hematological variables and serum analytes. These effects included hematocrit, differential leukocyte ratio, renal function biomarkers (BUN, creatinine), protein profiles, and selected electrolytes. During the study period, the temporal fluctuations observed in these variables probably reflect physiological adaptations or inherent biological variations that occurred within the animals. The hematocrit percentage was higher (*p* < 0.05) at D90 compared to D0. Neutrophil proportions were elevated (*p* < 0.05) at D90 and D45 relative to D0, whereas lymphocyte and monocyte percentages were greater (*p* < 0.05) at D0 and D45 than at D90. BUN levels peaked (*p* < 0.05) at D45, followed by D90, with the lowest levels observed at D0. Creatinine concentrations were similarly higher (*p* < 0.05) at D45 and D90 versus D0. Regarding protein profiles, total protein increased (*p* < 0.05) at D90 and D0 compared to D45. Albumin levels at D90 and D45 surpassed (*p* < 0.05) those of D0. In contrast, globulin concentrations were highest (*p* < 0.05) at D0, followed by D90, with D45 exhibiting the lowest values. Alkaline phosphatase activity peaked (*p* < 0.05) at D45, with lower levels observed at D0 and D90. Sodium concentrations were highest (*p* < 0.05) at D90 and reduced at D0 and D45. Carbon dioxide levels followed the order (*p* < 0.05): D0 > D45 > D90. Calcium exhibited maximum concentrations (*p* < 0.05) at D45, followed by D90, with the lowest concentration at D0. Finally, both magnesium and phosphorus increased (*p* < 0.05) at D45 and D90 relative to D0.

As presented in Table 2, the CBD inclusion in the diet of finishing goats did not modify growth performance, carcass, or non-carcass characteristics. Final body weight, weight gain, average daily gain, and carcass weight were not significantly different among the treatment groups (*p* > 0.05), indicating that CBD up to 0.3 mL/30 kg body weight did not have a detrimental effect on the overall growth and carcass yield of the animals. Similarly, dressing percentage, rib-eye area, and most carcass and non-carcass composition traits were not affected by the dietary treatments (*p* > 0.05). The only significant difference observed was in the percentage weight of hind shanks, which was lower in the T4 group than in the T1 and T2 groups (*p* = 0.041); however, this difference was relatively small and may not have practical implications. Regarding non-carcass characteristics, most variables, such as the percentage of the head, tail, foreshank, liver, heart, lungs and trachea, kidney, spleen, penis and testes, stomach, intestine, fat, blood, and GI content did not differ significantly among the treatment groups (*p* > 0.05).

The ultimate pH (Table 3) of the meat was not affected by the dietary treatments (*p* > 0.05), indicating that the CBD inclusion did not have a detrimental effect on post-mortem muscle metabolism. There was no significant difference in L* and b* values (*p* > 0.05). The a* and C* values were significantly higher in the T3 and T4 groups compared to the T2 group (*p* < 0.05). However, the hue angle (h*) showed the opposite trend (*p* = 0.003). These results suggest that the inclusion of higher levels of CBD (0.2 and 0.3 mL) may have improved the redness and overall color intensity of the meat. CBD did not affect the water-holding capacity of the meat. There was no significant difference in the percentages of drip loss and cooking loss (*p* > 0.05). Shear force and work of shear values were significantly affected by the dietary treatments. The T1 and T3 groups had significantly higher shear force values compared to the T2 and T4 groups (*p* < 0.001). Similarly, the value of the shear work was significantly higher in the T1 group compared to the T3 and T4 groups (*p* < 0.001), suggesting that CBD may have resulted in a softer texture of the meat. For the TPA analysis, the hardness and gumminess values were significantly lower in the T3 and T4 groups than in the T1 and T2 groups (*p* < 0.001), further confirming the potential tenderness-enhancing effect of CBD inclusion. Springiness values were also increased (*p* < 0.05). The cohesiveness and adhesion values were not modified by the dietary treatments (*p* > 0.05).

As shown in Table 4, the moisture content in goat meat from treatments T1 and T2 was significantly lower (*p* < 0.05) than that from treatments T3 and T4. In contrast, the protein content (g/kg DM) showed a significant increase (*p* < 0.05) in the latter treatments, while the fat content significantly decreased. Additionally, the protein percentage and ash content did not show significant differences across the treatments (*p* > 0.05).

Values for Sensor 1 (Table 5) were significantly elevated (*p* = 0.006) in the T3 and T4 groups compared with T1 and T2. Conversely, no treatment differences (*p* > 0.05) were detected for Sensors 2, 5, 6, and 7. An increasing trend from T1 to T4 was evident for Sensor 3, suggesting that a higher inclusion of hemp-derived CBD may have enhanced the concentrations of organic solvent vapor in meat. Sensor 4, which is primarily responsive to methyl mercaptan and trimethylamine, exhibited significantly higher signals (*p* = 0.007) in T4 compared to T1–T3. For Sensor 8, which detects ammonia, the T3 values were significantly higher (*p* = 0.048) than those of T1, while T2 and T4 were intermediary and did not differ from the other groups.

## 4. Discussion

The observed increase in WBC counts, particularly in the T2 (17.71 × 10^9^/L) and T4 (17.56 × 10^9^/L) groups, suggests a potential immunomodulatory effect of CBD supplementation in goats. This finding is confirmed by previous studies that demonstrated the anti-inflammatory and immunoregulatory properties of CBD [21,22]. WBC values are closely aligned with those reported in goats treated with fresh hemp leaves [6]; however, the WBCs in this study exceeded the reference ranges (4–13) reported in the literature [6,23,24,25,26]. Higher levels of total protein and globulin in the higher dose may indicate immune response modulation [12] or changes in protein metabolism induced by CBD supplementation. In this study, control samples (0 mL), along with T2 (0.1 mL) and T3 (0.2 mL) treatments, presented values within the normal WBC range; however, the T4 treatment (0.3 mL) induced a significant increase compared to the established normal values [25,27]. The current evidence is inadequate to confirm a direct correlation between CBD supplementation and high levels of these proteins, as all groups received the same feed; consequently, further empirical studies are needed. In contrast, the serum protein profiles of subjects administered with CBD oil [28] and those treated with fresh hemp leaves [6] did not show significant differences. Alterations in serum sodium and carbon dioxide concentrations attributable to CBD supplementation might have a modulatory role in electrolyte equilibrium and acid–base homeostasis in animals. This observation is similar to goats that were fed fresh hemp leaves, which exhibited reduced blood electrolyte levels compared to the control group [6]; however, the findings stand in stark contrast to those documented in [28], where non-significant fluctuations were observed in these variables after CBD administration. The temporal variations observed in various serum and hematologic biochemical indices, including hematocrit, differential leukocyte counts, markers of renal function, protein ranges, and electrolyte levels, underscore the intrinsic dynamism of these physiological parameters. Such changeability may be indicative of adaptive physiological responses or innate biological variability within the goats subjected to CBD throughout the investigation.

Dietary incorporation of hemp-derived CBD in goats did not substantially alter the key indicators of growth performance or the carcass and non-carcass characteristics evaluated. Weight gain and average daily gain were approximately 14.8 kg and 164.5 g and were not adversely affected by treatments. Another study found that despite comparable body weights across treatments, a linear decrease in average daily gain was observed with increasing dietary inclusion of hemp seed meal up to 33%, suggesting a negative impact on the growth performance of meat goats [29]. The weight gain observed in this study is consistent with previous findings [30] that indicate CBD oil supplementation at levels of 0.5 or 1.5 mg/kg does not affect body weight or body condition scores in animals. Similarly, animals fed full-fat hemp seed [31] demonstrated comparable performance. In quails, growth performance variables were negatively affected when hemp seed oil was substituted for soybean oil in their diets [32]. However, broiler chickens supplemented with hemp seed meal exhibited a positive growth response [33]. Therefore, the effects of hemp-derived CBD oil on the growth performance of animals remain equivocal, with findings exhibiting substantial variability depending on the concentration administered and the species studied. Although some studies report positive impacts on growth performance, others show no effect or even adverse effects.

Earlier investigation suggests that CBD may possess anti-inflammatory, antioxidant, and anxiolytic properties, which could influence animal health and well-being [12]. In the current study, dietary CBD inclusion had nonsignificant effects on dressing percentage and most carcass composition variables, indicating minimal impact on carcass yield and the proportions of various carcass components. The only exception was a slightly lower hind shank percentage observed in the group that received the highest CBD level; however, this difference was relatively minor and unlikely to have practical implications for carcass quality or value. Similar findings were reported by [31,34]. In addition, Gurung et al. [35] observed no adverse effects on carcass characteristics when goats were fed a 30% hemp seed diet. 

Meat pH is crucial for determining meat quality attributes such as color, water-holding capacity, and tenderness [13,36]. The CBD inclusion in the diet of finishing goats did not significantly affect the pH_u_ of the meat, indicating that post-mortem muscle metabolism and meat acidification processes were not compromised by the dietary treatments. This result aligns with previous studies on hemp in goats and cows [6,37]. Moreover, studies have demonstrated that meat redness is lower in animals fed fresh hemp leaves or hemp seeds compared to those in non-supplemented or soybean meal groups [6,37]. Particularly, the current incorporation of higher levels of CBD resulted in improved meat redness. The a* value exhibited a noticeable 17.94% reduction in the T2 group, while the T3 and T4 groups displayed marked increases of 14.51% and 21.97% relative to the control. This divergent response pattern was mirrored in the C* parameter, where the T2 group exhibited a 17.4% reduction, contrary to substantial increases of 11.1% and 20.6% in the T3 and T4 groups. The observed effects may be attributed to the putative antioxidant capacities of hemp-derived CBD [12,38,39,40], which could stabilize myoglobin pigments and inhibit their oxidation, thus conserving the preferred red color of the meat. 

A study on transported goats fed fresh hemp leaves showed that their meat had lower shear force than the control but these results were not statistically different [6]. This is consistent with the findings in cows, where hemp seed supplementation does not have a significant influence on the tenderness of the meat [37]. Surprisingly, our CBD inclusion, particularly at levels of 0.2 and 0.3 mL, exhibited a tenderness-enhancing effect on the meat. The shear force, work of shear, hardness, and gumminess values were significantly lower in these treatment groups, indicating improved tenderness and a softer meat texture. The shear force exhibited a substantial 50.94% decrease in the T2 group, whereas the T3 and T4 groups displayed more modest reductions of 8.75% and 34.41%, respectively. The work of shear of the T2 group revealed a 16.39% reduction, contrasted with more pronounced reductions of 24.33% and 47.09% in the T3 and T4 groups. The ameliorative impact observed may be attributable to the modulatory influence of CBD on the structure of muscle fibers, the activity of proteolytic enzymes, or the muscle metabolism [41,42], culminating in the augmented degradation of myofibrillar proteins and consequent tenderization of the meat. Furthermore, CBD may influence muscle lipid profiles by modulating fatty acid transporters and inhibiting de novo lipogenesis. These results may potentially affect muscle structure and function [43]. 

Previous studies of hemp by-products, such as hemp seed oil and leaves, demonstrated the potential to reduce odor in meats [6]. Hemp leaves may decrease meat odor because of their phenolic compounds with antioxidant properties that inhibit lipid oxidation [38,44]. Dietary incorporation of CBD reduced the formation of spoilage volatile organic compounds (VOCs), including alcohols, trimethylamine, and pentanoic acid, in stressed birds [45]. Moreover, CBD supplementation causes fatty acid degradation followed by a decrease in the spoilage VOC levels [45]. The current inclusion of higher levels of CBD in the diet of finishing goats appeared to influence the odor profile of the meat; this was evident from significant differences observed in the values of specific sensors used for odor analysis. Sensor 1, sensitive to gaseous species such as butane, methane, and propane, exhibited a striking 52.04% and 70.41% increase in responses for the T3 (0.2 mL) and T4 (0.3 mL) CBD groups compared to the control (T1), suggesting a significant elevation in the presence of these compounds. Similarly, an increasing trend was observed for Sensor 3, which detects organic solvent vapors, as there were incremental increases of 18.16%, 33.50%, and 52.76% in T2, T3, and T4 relative to the control, implying a dose-dependent effect of CBD on these volatile species. Sensor 4, primarily responsive to methyl mercaptan and trimethylamine, displayed a substantial 78.26% elevation in the T4 group, whereas T2 exhibited a 66.67% reduction, highlighting the intricate interplay between CBD levels and the volatile profile. Finally, Sensor 8, which is sensitive to ammonia, revealed a 6.81, 13.36, and 5.37% increase in responses for the T2, T3, and T4 groups compared with the control, indicating a potential modulation of ammonia-or-related compounds by specific CBD concentrations. These findings underscore the potential impact of higher levels of CBD inclusion on the presence of these VOCs in meat, although the specific compounds contributing to this trend were not identified in this study.

A potential limitation of this study is its small sample size, which might hinder the generalizability of the findings. Moreover, sarcomere length, myofibrillar fragmentation index (MFI), and collagen solubility were not included in this study, which could have provided valuable insights into the structural properties of the meat. However, the limited possibility of identifying individual volatile compounds emerging from meat restricts the general knowledge of the factors underlying the overall flavor and aroma of meat.

## 5. Conclusions

Dietary supplementation of hemp-derived cannabidiol (CBD) at a dose of 0.3 mL/30 kg BW in goats for 90 days was shown to improve meat tenderness and reduce fat content without adverse effects on growth performance and carcass traits. We recommend short-term feeding to maintain the physiological health of goats. Future studies should focus on refining these recommendations and investigating the long-term effects of dietary CBD consumption.

## Figures and Tables

**Table 1:** Effects of hemp-derived CBD on the blood variables of goats.

Variables	Treatments (A)				Time (B)			SEM	*p*-Values		
	T1	T2	T3	T4	Day 0	Day 45	Day 90		A	B	A × B
White blood cells (×10^9^/L)	11.73 ^b^	17.71 ^a^	12.76 ^b^	17.56 ^a^	16.28	12.99	15.54	0.741	0.009	0.103	0.504
Hemoglobin (mg%)	9.73	10.08	9.63	8.66	8.96	9.81	9.81	0.219	0.221	0.112	0.149
Hematocrit (%)	29.27	31.45	30.82	29.86	27.50 ^b^	30.75 ^ab^	32.79 ^a^	0.551	0.393	<0.001	0.637
Neutrophil (%)	48.50	54.50	55.42	59.67	41.87 ^b^	57.56 ^a^	64.13 ^a^	2.048	0.187	<0.001	0.600
Lymphocyte (%)	48.83	42.75	41.67	37.83	53.44 ^a^	40.31 ^ab^	34.56 ^b^	1.966	0.185	<0.001	0.937
Eosinophil (%)	0.75	1.33	2.17	1.17	2.00	0.94	1.13	0.245	0.452	0.056	0.094
Monocyte (%)	0.83	1.42	0.75	1.33	1.88 ^a^	1.19 ^a^	0.19 ^b^	0.218	0.542	0.007	0.313
Blood urea nitrogen (mg%)	14.18	14.06	13.98	15.24	11.23 ^b^	16.90 ^a^	14.96 ^ab^	0.542	0.792	<0.001	0.241
Creatinine (mg%)	0.96	0.91	0.89	1.05	0.84 ^b^	1.01 ^a^	1.02 ^a^	0.026	0.126	0.005	0.384
Protein total (mg%)	6.13 ^b^	6.54 ^b^	6.31 ^b^	7.22 ^a^	6.63 ^a^	6.17 ^b^	6.86 ^a^	0.094	0.000	0.001	0.448
Albumin (mg%)	3.09	3.14	3.03	3.14	2.52^b^	3.36^a^	3.42^a^	0.069	0.779	<0.001	0.397
Globulin (mg%)	3.07 ^b^	3.40 ^b^	3.28 ^b^	4.05 ^a^	4.12 ^a^	2.79 ^c^	3.44 ^b^	0.107	0.000	<0.001	0.172
Alkaline phosphatase (IU/L)	365.67	281.58	398.33	373.58	289.00^b^	469.69^a^	305.69^b^	29.753	0.505	0.026	0.418
Sodium (mmol/L)	148.97 ^a^	147.22 ^b^	146.39 ^b^	146.49 ^b^	146.70 ^b^	146.51 ^b^	148.59 ^a^	0.313	0.007	0.002	0.078
Potassium (mmol/L)	4.52	4.86	4.47	4.44	4.56	4.51	4.64	0.057	0.206	0.423	0.249
Chloride (mmol/L)	108.09	110.79	109.82	99.63	108.13	107.11	106.01	2.110	0.272	0.917	0.290
CO_2_ (mg/dL)	23.43 ^a^	19.37 ^b^	22.08 ^a^	22.86 ^a^	22.63 ^b^	22.74 ^a^	20.44 ^b^	0.403	0.004	0.005	0.162
Calcium (mg/dL)	10.34	10.10	9.78	9.81	9.79 ^b^	10.30 ^a^	9.93 ^ab^	0.088	0.215	0.020	0.581
Magnesium (mg/dL)	2.77	3.20	3.11	2.95	2.42 ^b^	3.48 ^a^	3.13 ^a^	0.099	0.489	<0.001	0.997
Phosphorus (mg/dL)	7.70	8.17	7.88	8.05	6.50 ^b^	9.05 ^a^	8.30 ^ab^	0.247	0.912	<0.001	0.829

T1 (control diet); T2–4, diets with 0.1, 0.2, and 0.3 mL CBD/30 kgBW; SEM: standard error of the mean; BUN: blood urea nitrogen; ^a–c^ means lacking a common superscript differ significantly (*p* < 0.05).

**Table 2:** Effects of hemp CBD on growth, carcass, and non-carcass characteristics of goat.

Variables	Treatments				SEM	*p*-Values
	T1	T2	T3	T4		
Initial body weight (kg)	12.20	13.20	14.03	16.96	0.702	0.074
Final body weight (kg)	27.23	29.46	28.00	30.91	1.307	0.798
Weight gain (kg)	15.03	16.26	13.97	13.96	0.974	0.846
Average daily gain (g)	167.00	180.68	155.23	155.08	10.818	0.846
Carcass weight (kg)	14.95	12.92	18.15	16.14	1.206	0.586
Dressing percentage (%)	53.16	49.76	56.31	54.98	1.327	0.394
Rib-eye area (cm^2^)	11.90	10.12	14.28	13.04	0.887	0.483
Carcass composition (%CW)						
Neck	9.93	9.05	10.41	9.30	0.414	0.753
Shoulder and foreleg	23.00	22.68	22.61	21.96	0.308	0.781
Rack	9.67	10.62	10.80	11.08	0.369	0.671
Loin	7.61	6.52	6.85	7.34	0.291	0.661
Tenderloin	1.23	0.96	0.88	0.74	0.077	0.103
Leg and hindleg	26.80	28.63	26.90	27.56	0.464	0.586
Breast	6.87	7.57	8.13	9.41	0.537	0.481
Flank	8.37	9.19	7.57	7.94	0.272	0.151
Sternum	4.22	4.24	3.83	4.07	0.094	0.476
Trimming	2.30	0.55	2.03	0.62	0.537	0.644
Non-carcass composition (%FBW)						
Head	6.11	6.95	6.47	7.12	0.267	0.635
Tail	0.89	0.53	0.67	0.78	0.064	0.235
Foreshank	1.53	1.67	1.47	1.32	0.065	0.331
Hind shank	1.63 ^a^	1.76 ^a^	1.52 ^ab^	1.31 ^b^	0.069	0.041
Liver	2.13	2.35	2.08	2.00	0.084	0.624
Heart	0.28	0.49	0.54	0.51	0.045	0.117
Lungs and trachea	1.89	1.63	1.47	1.53	0.111	0.671
Kidney	0.36	0.28	0.17	0.27	0.040	0.519
Spleen	0.32	0.08	0.10	0.20	0.043	0.170
Penis and testes	1.14	1.02	1.50	1.25	0.088	0.299
Stomach	3.37	3.53	3.02	3.74	0.208	0.767
Intestine	2.86	3.64	4.51	2.41	0.408	0.323
Fat	0.92	1.97	2.98	5.24	0.677	0.074
Blood and GI content	24.29	24.86	17.85	18.12	1.637	0.279

T1 (control diet); T2–4, diet with 0.1, 0.2, and 0.3 mL CBD/30 kgBW; SEM: standard error of the mean; ^a,b^ means lacking a common superscript differ significantly (*p* < 0.05). CW: carcass weight; FBW: final body weight; GI: gastrointestinal tract.

**Table 3:** Effects of hemp-derived CBD on the meat quality of goat.

Variables	Treatments				SEM	*p*-Values			
	T1	T2	T3	T4		ANOVA	L	Q	C
pHu	5.64	5.69	5.64	5.63	0.012	0.230	0.419	0.182	0.160
Color									
L*	44.54	41.59	39.42	44.21	0.958	0.190	0.688	0.052	0.437
a*	15.02 ^ab^	12.33 ^b^	17.20 ^a^	18.32 ^a^	0.854	0.030	0.023	0.144	0.064
b*	7.07	6.03	6.70	8.10	0.367	0.262	0.251	0.111	0.755
C*	16.61 ^ab^	13.72 ^b^	18.46 ^a^	20.03 ^a^	0.908	0.049	0.036	0.134	0.109
h*	71.49 ^a^	73.60 ^a^	66.42 ^b^	67.46 ^b^	0.980	0.003	0.003	0.608	0.004
Water-holding capacity (WHC)									
Drip loss (%)	7.66	4.60	4.50	5.79	0.506	0.075	0.161	0.026	0.693
Cooking loss (%)	31.81	29.45	26.94	31.73	1.446	0.703	0.754	0.094	0.402
Shear values									
Shear force (g)	6142.71 ^a^	3013.33 ^b^	5605.21 ^a^	4028.96 ^b^	278.9	<0.001	0.024	0.035	<0.001
Work of shear (mJ)	635.63 ^a^	531.46 ^ab^	480.97 ^b^	336.29 ^c^	26.24	<0.001	<0.001	0.591	0.383
Texture profile analysis (TPA)									
Hardness (g)	3855.56 ^a^	3157.69 ^a^	1021.88 ^b^	1441.31 ^b^	307.0	<0.001	<0.001	0.244	0.068
Cohesiveness	0.56	0.53	0.56	0.51	0.018	0.791	0.535	0.802	0.451
Springiness (mm)	2.61 ^b^	2.80 ^ab^	3.03 ^a^	3.11 ^a^	0.069	0.034	0.004	0.689	0.754
Adhesion (mJ)	0.07	0.03	0.02	0.06	0.010	0.086	0.602	0.013	0.853
Gumminess	2144.81 ^a^	1692.27 ^a^	587.50 ^b^	714.91 ^b^	170.1	<0.001	<0.001	0.271	0.114
Chewiness	5698.38 ^a^	4903.61 ^a^	1779.11^b^	1995.39 ^b^	474.2	0.001	<0.001	0.508	0.103

T1 (control diet); T2–4, diet with 0.1, 0.2, and 0.3 mL CBD/30 kgBW; SEM: standard error of the mean; ANOVA: analysis of variance; L: linear effect; Q: quadratic effect; C: cubic effect; ^a–c^ means lacking a common superscript differ significantly (*p* < 0.05). L* represents lightness (0 = black, 100 = white); a* represents the redness (−a = green, +a = red); b* represents the yellowness (−b = blue, +b = yellow); C* represents chroma or color saturation; h* represents the hue angle.

**Table 4:** Effects of hemp CBD on the chemical composition of meat.

Variables	Treatments				SEM	*p*-Values			
	T1	T2	T3	T4		ANOVA	L	Q	C
Moisture (%)	74.53 ^b^	74.73 ^b^	75.69 ^a^	75.91 ^a^	0.197	0.002	<0.001	0.954	0.120
Protein (%)	21.72	21.51	21.52	21.75	0.080	0.663	0.889	0.238	0.997
Protein (g/kg DM)	852.54 ^b^	851.19 ^b^	885.22 ^a^	903.01 ^a^	7.519	0.005	0.001	0.279	0.199
Fat (%)	2.98 ^a^	2.91 ^a^	1.65 ^b^	1.42 ^b^	0.241	<0.001	<0.001	0.699	0.034
Fat (g/kg DM)	120.76 ^a^	114.98 ^a^	67.65 ^b^	58.73 ^b^	9.062	0.001	<0.001	0.856	0.067
Ash (%)	0.90	1.03	1.20	1.05	0.045	0.103	0.115	0.103	0.323
Ash (g/kg DM)	35.45	40.75	49.38	43.39	1.983	0.061	0.042	0.097	0.218

T1, control diet; T2–4, diet with 0.1, 0.2, and 0.3 mL CBD/30 kgBW; SEM: standard error of the mean; ANOVA: analysis of variance; L: linear effect; Q: quadratic effect; C: cubic effect; DM: dry matter; ^a,b^ means lacking a common superscript differ significantly (*p* < 0.05).

**Table 5:** Effects of hemp-derived CBD on the meat odor of goat.

Variables	Treatments				SEM	*p*-Values			
	T1	T2	T3	T4		ANOVA	L	Q	C
Sensor 1	0.196 ^b^	0.177 ^b^	0.298 ^a^	0.338 ^a^	0.028	0.006	0.001	0.283	0.090
Sensor 2	0.220	0.184	0.219	0.278	0.019	0.161	0.110	0.105	0.708
Sensor 3	0.815 ^d^	0.963 ^c^	1.088 ^b^	1.245 ^a^	0.062	0.000	<0.001	0.908	0.754
Sensor 4	0.069 ^b^	0.023 ^b^	0.060 ^b^	0.123 ^a^	0.015	0.007	0.014	0.005	0.384
Sensor 5	0.268	0.234	0.219	0.276	0.020	0.626	0.950	0.227	0.738
Sensor 6	0.220	0.205	0.227	0.273	0.015	0.219	0.098	0.197	0.911
Sensor 7	0.217	0.156	0.184	0.238	0.017	0.155	0.411	0.043	0.564
Sensor 8	2.628 ^b^	2.807 ^ab^	2.979 ^a^	2.769 ^ab^	0.059	0.048	0.098	0.025	0.271

T1, control diet; T2–4, diet with 0.1, 0.2, and 0.3 mL CBD/30 kgBW; SEM: standard error of the mean; ANOVA: analysis of variance; L: linear effect; Q: quadratic effect; C: cubic effect; ^a–d^ means lacking a common superscript differ significantly (*p* < 0.05).

## Data Availability

Data are contained within the article.
